# circ-IARS depletion inhibits the progression of non-small-cell lung cancer by circ-IARS/miR-1252-5p/HDGF ceRNA pathway

**DOI:** 10.1515/med-2022-0613

**Published:** 2023-01-11

**Authors:** Jinhua Yang, Chunping Yang, Ping Li

**Affiliations:** Department of Thoracic and Cardiovascular Surgery, Zigong First People’s Hospital, No. 178, Tongda South Street, Ziliujing District, Zigong, Sichuan, China; Department of Thoracic and Cardiovascular Surgery, Zigong First People’s Hospital, Zigong, Sichuan, China

**Keywords:** circ-IARS, miR-1252-5p, HDGF, NSCLC, exosome

## Abstract

This study aims to explore the role and mechanism of circ-IARS in non-small-cell lung cancer (NSCLC) progression. Expression of circ-IARS, microRNA (miR)-1252-5p, and hepatoma-derived growth factor (HDGF) was measured by real-time quantitative PCR and western blotting. The interactions among circ-IARS, miR-1252-5p, and HDGF were determined by dual-luciferase reporter assay and RNA immunoprecipitation. Cell behaviors were measured by 3-(4,5-dimethylthiazol-2-yl)-2,5-diphenyltetrazolium bromide (MTT), 5-ethynyl-2′-deoxyuridine (EdU) assay, flow cytometry, scratch wound assay, and transwell assay, and validated in *in vivo* xenograft model. Exosomes were isolated using commercial kit, and the expression and functions of exosomal circ-IARS (exo-circ-IARS) were analyzed as described above. Results showed that the expression of circ-IARS was upregulated in NSCLC cells, NSCLC tissues, and serum exosomes from NSCLC patients. circ-IARS exhaustion antagonized cell proliferation, cell cycle progression, migration, and invasion and promoted apoptosis in NSCLC. Molecularly, circ-IARS could sponge miR-1252-5p to modulate the expression of the downstream gene HDGF. In addition, miR-1252-5p downregulation attenuated circ-IARS exhaustion-mediated effects in H1299 and A549 cells. MiR-1252-5p mimic-induced effects were relieved by increasing HDGF expression in H1299 and A549 cells. Exo-circ-IARS promoted H460 cell proliferation, migration, and invasion and inhibited cell apoptosis. Silencing circ-IARS retarded tumor growth of NSCLC cells *in vivo*. Thus, circ-IARS, secreted by exosomes, was a novel oncogene in NSCLC and regulated the malignant development of NSCLC cells via circ-IARS/miR-1252-5p/HDGF competing endogenous RNA regulatory axis.

## 1 Introduction

Lung cancer is one of the most common causes of cancer incidence and cancer death among men and women in the United States and China [1,2]. Non-small-cell lung cancer (NSCLC) accounts for approximately 85% of all lung cancer cases [3], and other cases are small-cell lung cancer. NSCLC carries a 5 year survival rate of 15% [4], and early detection could offer a favorable prognosis with a 5 year survival rate of 92% [5]. However, more than 75% of NSCLC patients are diagnosed in advanced stage or metastasis [4,6]. The current treatment approaches revolve around targeting oncogenes, and small interfering RNA (siRNA) therapy combined with nanosystems possess extraordinary potential to address the clinical needs for higher efficacy [7]. Due to the stable trend of lung cancer in China [2], it is especially important and imperative to identify novel biomolecules guiding the early detection of NSCLC and the effective treatment.

Exosomes are nano-sized particles secreted from most cells that communicate with surrounding environment by carrying and transferring informative cargos, such as protein, DNA, and RNA [8]. Increasing evidence implies that exosomes participate in physiological and pathological processes, including carcinogenesis [9]. In NSCLC, liquid biopsies have the potential as tools for diagnosis, prognosis, and prediction of response to therapy, as well as disease monitoring [10]. Moreover, exosomes are universally present in the blood and other bodily fluids [9]; their stability in blood and their similarity to the cells of origin make tumor-derived exosomes as candidate biomarkers and immunological effects for lung cancers [8,11,12].

Circular RNAs (circRNAs) are a type of non-coding RNAs that form a covalently closed continuous loop, and circRNAs are enriched and stable in exosomes [13]. Functionally, exosomal circRNAs are involved in malignant behaviors of cancer cells, such as proliferation, invasion, metastasis, and chemoresistance [13]. Previous research shed light on genome-wide transcriptome profiling of coding genes, long noncoding RNAs, and circRNAs in patients with lung adenocarcinoma (LUAD) [14], the major histological type of NSCLC. The circRNA hsa_circ_0006702 is derived from the isoleucyl-tRNA synthetase 1 (IARS) gene, thus termed circ-IARS, which is 3.6-fold higher in LUAD tumors compared with paired nontumor tissues [14]. However, the expression of circ-IARS remains undisclosed in the serum or serum exosome of NSCLC patients; moreover, whether this abnormally expressed circRNA plays important roles in biological processes of NSCLC is unknown. Thus, in this study, we intended to explore the role of circ-IARS and exosomal circ-IARS (exo-circ-IARS) in NSCLC cell growth, migration, and invasion.

CircRNAs are crucial molecules in the regulation of tumorigenesis, progression, invasion, and metastasis in lung cancers via classic circRNA/microRNA (miRNA)/messenger RNA (mRNA) regulatory network [15]. The latent circRNA/miRNA/mRNA interactions have emerged as the mechanism of exosomal circRNAs during their functions in promoting or inhibiting cancer genesis and progression [16,17]. miR-1252-5p is a miRNA that has not been studied until recent years, and hepatoma-derived growth factor (HDGF) is a novel jack-of-all-trades in cancer including NSCLC [18,19]. Through prediction of Circinteractome and StarBase online databases in the preliminary experiment, we found that circ-IARS contained the binding sites for miR-1252-5p and miR-1252-5p potentially targeted HDGF. Based on the prediction, we further analyzed whether the competing endogenous RNA (ceRNA) mechanism related to NSCLC progression involved circ-IARS, miR-1252-5p, and HDGF.

## 2 Materials and methods

### 2.1 Cell culture and cell transfection

Bronchial epithelium cell line BEAS-2B (No. KCB200922YJ), NSCLC cell lines including H1299 (No. 25803), A549 (No. 10185), and H460 (No. 30177), and human embryonic kidney (HEK) 293T (No. KCB200744YJ) were from Kunming Cell Bank (Kunming, China) or Korean Cell Line Bank (Seoul, Korea). These cells were cultured in Roswell Park Memorial Institute (RPMI)-1640 (No. M31050; R&D systems, Minneapolis, MN, USA) or Dulbecco’s Modified Eagle Medium (DMEM, M22650; R&D systems) supplemented with 10% fetal bovine serum (No. S11550H; R&D systems). H1299 and A549 cells were exogenously transfected with small interfering RNAs (siRNAs) targeting circ-IARS (si-circ-IARS_1 and _2), miR-1252-5p mimic or inhibitor, and pcDNA3.1/Zeo(+) vector (pcDNA; EK-Bioscience) carrying HDGF (HDGF), and pCD-ciR vector (EK-Bioscience) carrying circ-IARS (circ-IARS), as well as pSilencer 4.1-CMV puro vector (pSilencer) carrying short hairpin RNA (shRNA) targeting circ-IARS (sh-circ-IARS). The siRNA targeting negative control (si-NC), NC mimic or inhibitor, and empty pcDNA and pCD-ciR, as well as pSilencer carrying sh-NC (sh-NC) served as negative controls. The transfection was performed using RNAifectin Transfection Reagent (Applied Biological Materials, Richmond, Canada) following the protocols. The oligo sequences are summarized in Table 1. Post-transfection for 36 h, the cells were harvested for RNA (/protein) isolation or functional analysis.

**Table 1:** Sequences of oligos and primers

Name	Sequence
si-circ-IARS_1	5′–UUAGAGGAGUGUCUGAUCUGCdTdT–3′
si-circ-IARS_2	5′–AUUAGAGGAGUGUCUGAUCUGdTdT–3′
sh-circ-IARS	5′–UUAGAGGAGUGUCUGAUCUGC–3′
miR-1252-5p mimic	5′-AGAAGGAAAUUGAAUUCAUUUA-3′
miR-1252-5p inhibitor	5′–TAAATGAATTCAATTTCCTTCT–3′
si-NC	5′–UUCUCCGAACGUGUCACGUdTdT–3′
sh-NC	5′–UUCUCCGAACGUGUCACGU–3′
NC mimic	5′–ACGUGACACGUUCGGAGAATT–3′
NC inhibitor	5′–CAGUACUUUUGUGUAGUACAA–3′
circ-IARS (125nt)	Forward primer 5′–TTACAGACCGGTGGATCCTG–3′
	Reverse primer 5′–TGTTCTCCACTCGCACAAAC–3′
miR-1252-5p (75nt)	F 5′–ACACTCCAGCTGGGAGAAGGAAATTGAATT–3′
	R 5′–TGTCGTGGAGTCGGCAATTC–3′
HDGF (79nt)	Forward primer 5′–AGTACAAATGCGGGGACCTG–3′
	Reverse primer 5′–TCAGGCATCTCGTCAATCCG–3′
β-actin (104nt)	Forward primer 5′–CTTCGCGGGCGACGAT–3′
	Reverse primer 5′–CCACATAGGAATCCTTCTGACC–3′
U6 (87nt)	Forward primer 5′–CGCTTCGGCAGCACATATAC–3′
	Reverse primer 5′–TTCACGAATTTGCGTGTCATC–3′

### 2.2 Real-time quantitative PCR (RT-qPCR)

Total RNAs from tissues or cells were isolated with TRIzol reagent (Invitrogen, Carlsbad, CA, USA) referring to the supplier’s direction. These RNAs were reversely transcribed into the first-strand complementary DNA (cDNA) using miScript reverse transcription kit (Qiagen, Nasdaq, NK, USA) and then amplified using miScript SYBR-Green PCR kit (Qiagen) and corresponding primer pairs targeting circ-IARS, miR-1252-5p, or HDGF. Expression of actin beta (β-actin; for circ-IARS and HDGF) and U6 small nuclear RNA (U6; for miR-1252-5p) was used as the internal controls. The 2^−ΔΔCt^ method was used to evaluate the expression levels. The primer sequences are exhibited in Table 1.

### 2.3 Cell proliferation assay

Cell proliferation was measured by 3-(4,5-dimethylthiazol-2-yl)-2,5-diphenyltetrazolium bromide (MTT) assay and 5-ethynyl-2′-deoxyuridine (EdU) assay using MTT Cell Proliferation and Cytotoxicity Assay Kit (Beyotime, Shanghai, China) and iClick^TM^ EdU Andy Fluor^TM^ 555 Imaging Kit (GeneCopoeia, Rockville, MD, USA). For MTT assay, H1299, A549, and H460 cells were re-inoculated in 96-well plates at 2,000 cells/well, and cultured for another 3 days. Post-cultivation, these cells were treated with 10 μL of MTT reagent (5 mg/mL) for 4 h at 1, 2, and 3 days, and then the formazan was dissolved in 100 μL of formazan reagent for 4 h. Optical density (OD) was examined at 490 nm on microplate reader (Molecular devices, Shanghai, China). For EdU assay, cells were labelled with 10 μM EdU and reacted with Andy Fluor488 azide (Component B); then, the labelled cells were subjected to Hoechst 33342 counterstain followed by observation under fluorescence microscopy. The EdU-positive rate was expressed as the percentage of EdU-positive cells to Hoechst-positive cells.

### 2.4 Cell cycle analysis and apoptosis assay by flow cytometry (FCM)

Cell cycle profile was evaluated by Propidium Iodide (PI; Beyotime) staining using Cell Cycle and Apoptosis Analysis Kit (Beyotime). 1 × 10^5^ H1299 or A549 cells were fixed in ice-cold 70% ethanol overnight and then incubated with 1× PI reagent supplemented with 1× RNase A for 30 min in the dark. DNA contents were examined and analyzed on flow cytometer (BD Biosciences, Franklin Lake, NJ, USA) and Modifit software (BD Biosciences). Cell death was determined using Annexin V-fluorescein isothiocyanate (FITC) Apoptosis Detection Kit (Beyotime). According to the protocols, 1 × 10^5^ H1299, A549, or H460 cells were dual-stained with 5 μL of Annexin V-FITC and 10 μL of PI for 20 min in the dark and then examined on flow cytometer (BD Biosciences). Apoptotic cells were calculated as the percentage of cells in Annexin V-FITC+/PI+ and Annexin V-FITC+/PI- quadrants.

### 2.5 Cell migration and invasion assays

To measure cell migration, scratch wounds were generated on the surface of 24-well plates by straightly moving a 100 μL of pipette across the center of every well. The remaining 5 × 10^4^ cells (H1299, A549, or H460) were cultured in serum-free medium, and wounds were photographed after wound healing for 0 and 24 h. Wound area was measured on ImageJ v1.52 software (National Institute of Health, Bethesda, MD, USA), and the percentage of wound closure was calculated.

Transwell (Corning, Corning, NY, USA) was utilized to measure cell invasion. First of all, transwell supports were coated with Matrigel (BD Biosciences); then, 5 × 10^4^ H1299, A549, or H460 cells were collected in serum-free medium and seeded in the upper chamber, and the lower chamber was filled with medium containing 10% fetal bovine serum. Transwell invasion assay was incubated at 37°C for 48 h, and invaded cells on the lower surface were fixed with 4% Paraformaldehyde Fix Solution (Beyotime) and stained with Crystal Violet Staining Solution (Beyotime) for 20 min. The stained cells were observed under a microscope (100×; Olympus, Tokyo, Japan).

### 2.6 Dual-luciferase reporter assay and RNA immunoprecipitation (RIP)

The algorithms of Circinteractome (https://circinteractome.nia.nih.gov/mirna_target_sites.html) and StarBase (http://starbase.sysu.edu.cn/agoClipRNA.php? source = circRNA) were applied for predicting the target miRNAs in circ-IARS sequence. StarBase (http://starbase.sysu.edu.cn/agoClipRNA.php?source = mRNA) also predicted the binding sites of miR-1252-5p in HDGF. The wild type (WT) of circ-IARS and 3′-untranslated region of HDGF (HDGF 3′UTR) were synthesized, annealed, and then inserted into the *Sac*I and *Hind*III sites of pMIR-REPORTER Luciferase vector (EK-Bioscience) at downstream of the stop codon of the gene for luciferase. Similarly, the mutant types (MUT) of circ-IARS and HDGF 3′UTR were generated with a mutant at the predicted miR-1252-5p-binding sites and then inserted into pMIR-REPORTER Luciferase vector (EK-Bioscience), as well. 5 × 10^4^ HEK293T cells in 24-well plates were co-transfected with the above luciferase vectors, miR-1252-5p (/NC) mimic, and pRL-TK vector (EK-Bioscience) using RNAifectin Transfection Reagent (Applied Biological Materials). After transfection for 48 h, *Firefly* and *Renilla* luciferase activities were tested using the Dual-Luciferase Reporter assay system (Promega, Madison, WI, USA), and *Renilla* was used as an internal control.

Magna RIP RNA-Binding Protein Immunoprecipitation Kit (Millipore, Billerica, MA, USA) was employed to analyze the interplay between circ-IARS and miR-1252-5p. H1299 and A549 cells were lysed in RIP Lysis Buffer, and the whole cell lysate was incubated with A/G magnetic beads pre-covered with anti-Argonaute 2 (anti-AGO2; ab186733, Abcam) or anti-immunoglobulin G (anti-IgG; ab182931, Abcam) overnight at 4°C with gentle rotation. The precipitated RNA–protein complexes on the beads were washed and treated with proteinase K for 30 min to obtain precipitated RNAs, which were further isolated in TRIzol reagent (Invitrogen) for RT-qPCR analysis. IgG was employed as a negative control, and the input functioned as a positive control.

### 2.7 Western blotting

Total proteins in tissues and cells were extracted by Radio Immunoprecipitation Assay (RIPA) Lysis Buffer (Beyotime). After measuring protein concentration using bicinchoninic acid assay (BCA) Protein Assay Kit (Beyotime), 30 μg protein samples were separated on sodium dodecyl sulfate-polyacrylamide gel electrophoresis (SDS-PAGE), transferred onto Immobilon-Nitrocellulose (NC) Transfer Membrane (Millipore), and probed with antibodies including Tumor Susceptibility 101 (TSG101), CD9, CD63, and β-actin. The β-actin was considered as the endogenous control. The antibodies are summarized in Table 2. Eventually, the protein signals were visualized by Immobilon ECL HRP substrate (Millipore) and analyzed on ImageJ v1.52 software (National Institute of Health).

**Table 2:** Antibodies used in western blotting

Antibody	No.	Dilution rate	Source
HDGF	#ab248221	1:1,000/1,100	Abcam
TSG101	#ab83	1:2,500	Abcam
CD63	#ab271286	1:1,000	Abcam
CD9	#ab263019	1:1,000	Abcam
Ki67	#ab15580	1:250	Abcam
β-actin	#ab8226	1:2,000	Abcam
Rabbit IgG (HRP)	#ab205718	1:50,000	Abcam
Mouse IgG (HRP)	#ab97023	1:20,000	Abcam

### 2.8 Tumorigenesis in nude mice

H1299 cells transfected with shRNAs were selected with 1 µg/mL puromycin (Invitrogen) for 4 weeks to generate stable sh-circ-IARS (/NC)-transfected cells. This animal experiment was approved by the Animal Care Committee of the Zigong First People’s Hospital. According to the Animal Research Reporting of *In Vivo* Experiments (ARRIVE) Guidelines and the Basel Declaration, 10 BALB/c nude mice (male; Beijing Vital River Laboratory Animal Technology Company, Beijing, China) were housed and subcutaneously injected with the above stably transfected H1299 cells. Briefly, 2 × 10^6^ cells in 100 μL of normal saline were injected into each mouse, and 5 mice were set in each group. The tumor volume was determined every 5 days during a month by measuring the tumor size (length and width) with Vernier caliper: 0.5 × length × width^2^. Tumor weight was examined on the last day by electronic scales. The xenograft tumor tissues were resected, paraffin-embedded, and stored. Total RNAs (/proteins) were isolated from these tumor tissues from nude mice, and immunohistochemistory (IHC) assay was used to measure the expression of HDGF and Ki67 *in situ*.

### 2.9 Clinical specimens from patients

The blood and tissue samples were collected from the Zigong First People’s Hospital. A total of 44 paired tumor tissues and adjacent non-tumor tissues were obtained from NSCLC patients during surgery and soaked in liquid nitrogen. Peripheral blood samples (3.5 mL) were from 22 NSCLC patients and 22 healthy people and put into anticoagulant-free tubes. Blood was centrifuged at 3,000 g for 10 min, and the supernatant was harvested as serum samples. Clinicopathological factors of NSCLC patients and healthy volunteers are presented in Table A1. All the experiments were performed in accordance with the Code of Ethics of the World Medical Association. All subjects signed the written informed consents and this study was supported by the Ethics Committee of Zigong First People’s Hospital. Later, tissue samples were used for total RNAs (/proteins) isolation, and serum samples were utilized for exosome isolation.

### 2.10 Exosomes isolation and treatment

GSTM Exosome Isolation Reagent for serum (Geneseed, Guangzhou, China) and GSTM Exosome Isolation Reagent for cell culture medium (Geneseed) were used for exosome isolation. The extraction processes were in strict accordance with the directions. Transmission electron microscope (TEM) was applied to examine the morphology of extracted exosomes according to the previously described process [20]. Total RNAs and proteins were isolated by TRIzol reagent (Invitrogen) and RIPA (Beyotime), respectively. Expression of the exosomal circ-IARS (exo-circ-IARS) was detected by RT-qPCR, and western blotting was used to detect the expression of exosome markers including TSG101, CD9, and CD63. The antibodies are summarized in Table 2. H1299 cells-based exosomes after circ-IARS or pCD-ciR vector transfection were termed as pCD-ciR-exo or circ-IARS-exo. H460 cells were co-cultured with exosomes for 48 h prior to functional experiments.

### 2.11 Statistical analysis

Distribution normality was evaluated using Shapiro–Wilk normality test. All the data were expressed as mean value ± standard deviation and analyzed using two-tailed Student’s *t* test and analysis of variance method with Tukey’s test on GraphPad Prism version 7 (GraphPad Software, La Jolla, CA, USA). It was considered as significant when *P* < 0.05. Spearman’s rank correlation analysis was used to obtain the bivariate correlations.


**Ethics approval and consent to participate**: The present study was approved by the ethical review committee of Zigong First People’s Hospital. Written informed consent was obtained from all enrolled patients.
**Consent for publication**: Patients agreed to participate in this work.

## 3 Results

### 3.1 Exhausting circ-IARS antagonized the malignant growth, migration, and invasion of NSCLC cells *in vitro*

**Figure 1:**
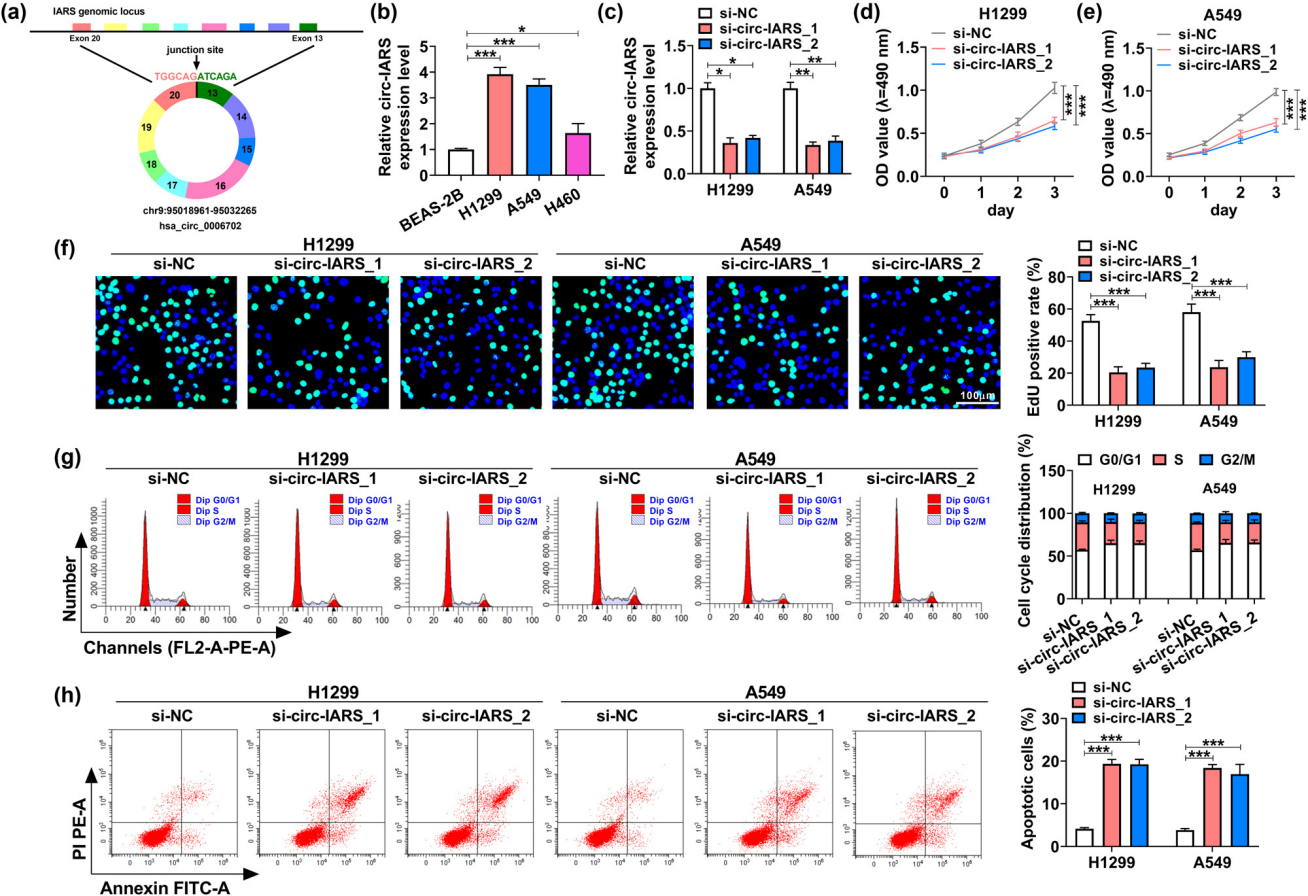
Exhausting circ-IARS could antagonize NSCLC cell growth. (a) Schematic diagram showing the back-splicing of exons 13–20 of the IARS gene generating the circRNA hsa_circ_0006702. (b and c) RT-qPCR is used to measure the relative circ-IARS expression level in (b) H1299, A549, H460, and BEAS-2B cells, and in (c) H1299 and A549 cells transfected with si-circ-IARS_1, si-circ-IARS_2, or si-NC. In transfected H1299 (d) and A549 cells (e), MTT assay monitored the OD value at 490 nm, (f) EdU assay determined the EdU-positive rate, (g and h) FCM method analyzed the cell cycle distribution (%) in G0/G1, S, and G2/M phases, and apoptotic cells (%). *P < 0.05, **P < 0.01, and ***P < 0.001.

**Figure 2:**
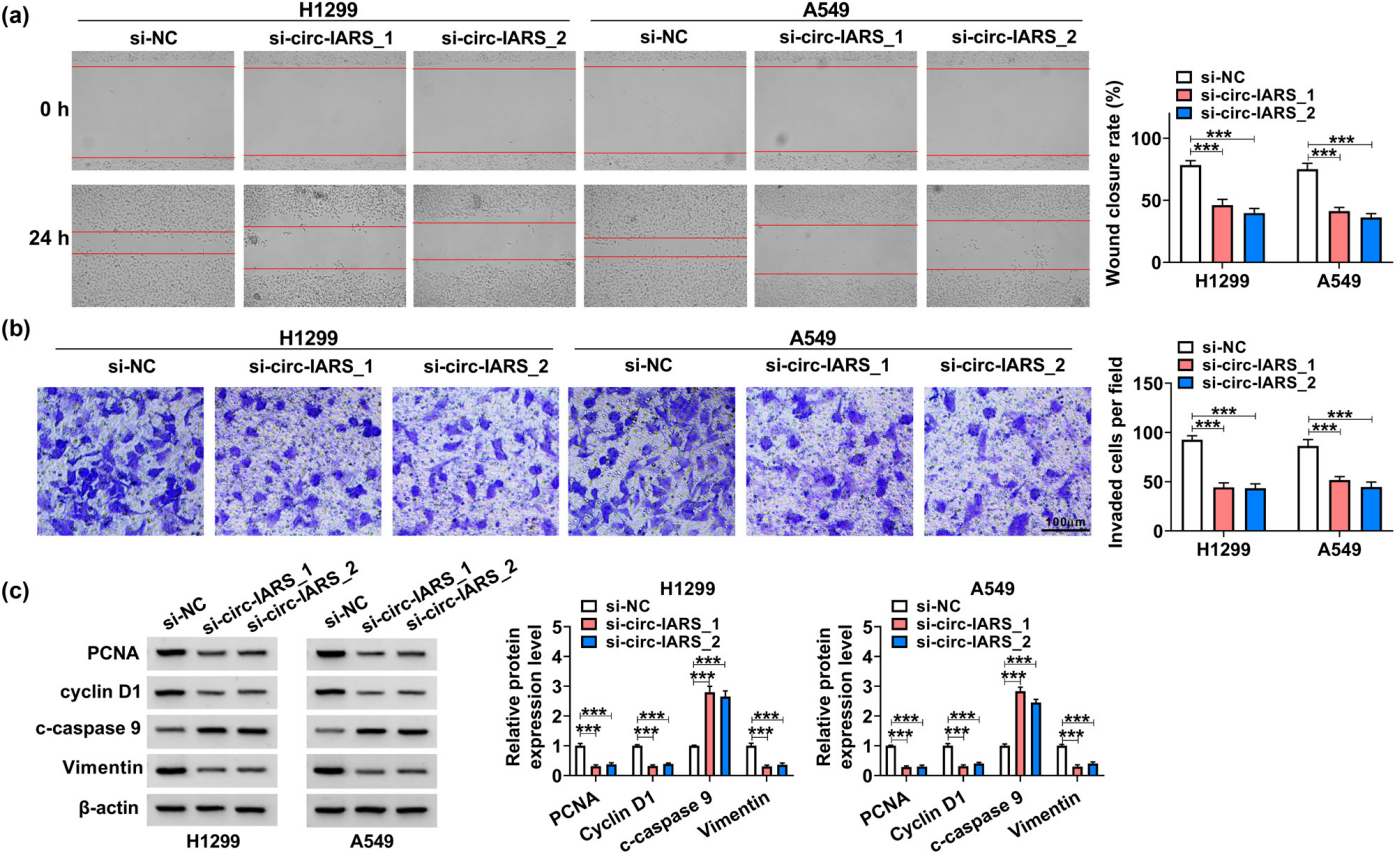
Exhausting circ-IARS could antagonize cell migration and invasion of NSCLC cells. (a) Scratch wound assay measured wound closure rate in H1299 and A549 cells after transfection. (b) Transwell assay examined the invaded cells per field (100×) after transfection. (c) Western blotting detected the relative protein expression levels of PCNA, cyclin D1, c-caspase 9, and vimentin in transfected cells, normalized to β-actin. ***P < 0.001.

**Figure A1:**
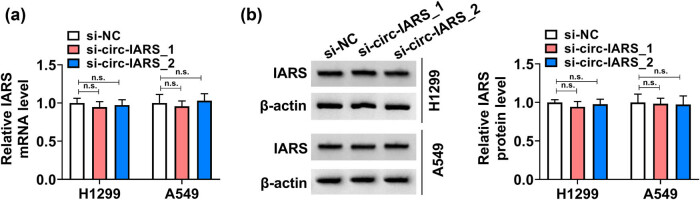
The effects of si-circ-IARS_1 and si-circ-IARS_2 on IARS mRNA and protein expression were analyzed by RT-qPCR (A) and Western blotting (B) in H1299 and A549 cells. ns: no significant difference.

circ-IARS was a circRNA derived from the exons 13–20 of the IARS gene (Figure 1a), and its expression level was higher in NSCLC cells (H1299, A549, and H460) than that in normal BEAS-2B cells (3.92 fold, 3.50 fold, and 1.64 fold, respectively, Figure 1b). Circ-IARS expression was dramatically downregulated by more than 50%, whereas IARS expression was not affected after transfection with si-circ-IARS_1 or si-circ-IARS_2 (Figure 1c and Figure A1), indicating that si-circ-IARS_1 and si-circ-IARS_2 were effective in reducing circ-IARS expression. si-circ-IARS (both _1 and _2) transfection reduced the proliferation of H1299 and A549 cells (Figure 1d and e), accompanied by a lower EdU-positive rate (Figure 1f). Cell cycle distribution in G0/G1 phase was increased, and cells in the S phase were decreased in H1299 and A549 cells after circ-IARS downregulation (Figure 1g). Meanwhile, apoptotic cells were highly induced (more than 4.42 fold) in circ-IARS-silenced H1299 and A549 cells (Figure 1h). Cell migration and invasion were abnormally inhibited by exhausting circ-IARS (Figure 2a and b). Circ-IARS silencing led to the upregulation of c-caspase 9 and the downregulation of PCNA, cyclin D1, and vimentin (Figure 2c). These results demonstrated that exhausting circ-IARS could antagonize the malignant development of NSCLC cells *in vitro*.

### 3.2 circ-IARS served as a ceRNA for miR-1252-5p to regulate HDGF in NSCLC cells

**Figure 3:**
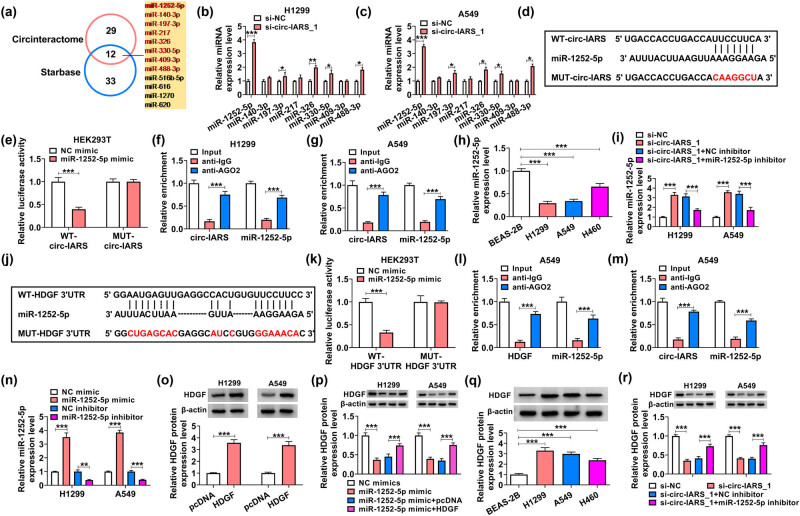
Circ-IARS served as a ceRNA for miR-1252-5p to regulate HDGF in NSCLC cells. (a) Circinteractome website and StarBase website together presented 12 miRNAs that were complementary to circ-IARS. (b and c) RT-qPCR detected the relative expression levels of 8 miRNAs in H1299 and A549 cells transfected with si-circ-IARS_1 or si-NC. (d) Schematic diagram showing the predicted binding sites between WT-circ-IARS and miR-1252-5p sequences. (e) Dual-luciferase reporter assay measured the relative luciferase activity of WT-circ-IARS reporter vector and MUT-circ-IARS reporter vector in HEK293T cells transfected with miR-1252-5p mimic or NC mimic. (f and g) RIP assay evaluated the relative enrichment of circ-IARS and miR-1252-5p by AGO2 or IgG in H1299 and A549 cells, normalized to the input of cells. (h and i) RT-qPCR measured the relative miR-1252-5p expression level in (h) BEAS-2B, H1299, A549, and H460 cells, and (i) H1299 and A549 cells transfected with si-NC, si-circ-IARS_1, si-circ-IARS_1 + miR-1252-5p inhibitor, or si-circ-IARS_1 + NC inhibitor. (j) Schematic diagram showing the predicted binding sites between WT-HDGF 3′UTR and miR-1252-5p sequences. (k) Dual-luciferase reporter assay measured the relative luciferase activity of WT-HDGF 3′UTR reporter vector and MUT-HDGF 3′UTR reporter vector in HEK293T cells transfected with miR-1252-5p mimic or NC mimic. (l and m) RIP assay evaluated the relative enrichment of HDGF and miR-1252-5p by AGO2 or IgG in H1299 and A549 cells, normalized to the input of cells. (n) RT-qPCR measured the relative miR-1252-5p expression level in H1299 and A549 cells transfected with miR-1252-5p mimic, miR-1252-5p inhibitor, NC mimic, or NC inhibitor. (o–r) Western blotting detected the relative HDGF protein expression in (o and p) H1299 and A549 cells transfected with HDGF vector, pcDNA vector, miR-1252-5p mimic, NC mimic, miR-1252-5p mimic + HDGF vector, or miR-1252-5p mimic + pcDNA vector, and (q) BEAS-2B, H1299, A549, and H460 cells, as well as (r) H1299 and A549 cells transfected with si-NC, si-circ-IARS_1, si-circ-IARS_1 + miR-1252-5p inhibitor, or si-circ-IARS_1 + NC inhibitor. *P < 0.05, **P < 0.01, and ***P < 0.001.

With bioinformatics algorithm prediction, Circinteractome and StarBase websites presented a total of 12 miRNAs that were potentially complementary to circ-IARS (Figure 3a). Among these miRNAs, 8 miRNAs were downregulated in NSCLC. Besides, miR-1252-5p was the most sensitive one (change, 3.83 fold and 3.54 fold) in response to circ-IARS deficiency in H1299 and A549 cells (Figure 3b and c). Thus, miR-1252-5p was employed as the promising target for circ-IARS. MUT-circ-IARS was generated according to the mutation of the predicted binding sequence in WT-circ-IARS (Figure 3d), and luciferase activity of WT-circ-IARS reporter vector was repressed by 60.00% in HEK293T cells with miR-1252-5p overexpression (Figure 3e). While the luciferase activity of MUT-circ-IARS vector was not affected on miR-1252-5p mimic transfection. Moreover, RIP assay showed a co-enrichment of circ-IARS and miR-1252-5p by AGO2 in both H1299 and A549 cells (Figure 3f and g). Thereby, miR-1252-5p was confirmed as one target for circ-IARS, and this miRNA was shown to be lowly expressed in three NSCLC cells (Figure 3h). Additionally, the promotion of circ-IARS exhaustion on miR-1252-5p could be partially canceled by co-transfection of miR-1252-5p inhibitor (Figure 3i).

Subsequently, the downstream target gene of miR-1252-5p was further explored. Here we investigated the relationship between miR-1252-5p and HDGF according to the potential binding sites (Figure 3j). Dual-luciferase reporter assay revealed that miR-1252-5p mimic impaired luciferase activity of WT-HDGF 3′UTR vector by 67.00% and failed to alter the luciferase activity of MUT-HDGF 3′UTR vector in HEK293T cells (Figure 3k). RIP assay revealed that miR-1252-5p and HDGF were synchronously enriched by AGO2 in both H1299 and A549 cells (Figure 3l and m). To abnormally express miR-1252-5p, H1299 and A549 cells were transfected with miR-1252-5p mimic or inhibitor (Figure 3n). Similarly, HDGF was exogenously overexpressed with a change of 3.57 fold and 3.37 fold after transfection with HDGF vector (Figure 3o). The expression of HDGF was inhibited in miR-1252-5p-upregulated or circ-IARS-downregulated H1299 and A549 cells (Figure 3p and r), but this low HDGF expression could be reversed by co-transfection of HDGF vector or miR-1252-5p inhibitor (Figure 3p and r). By the way, HDGF was highly expressed in NSCLC cells when compared with that in BEAS-2B cells (Figure 3q). The above results might indicate that circ-IARS segregated miR-1252-5p to modulate HDGF, suggesting a circ-IARS/miR-1252-5p/HDGF ceRNA axis in NSCLC.

### 3.3 There was a circ-IARS/miR-1252-5p/HDGF ceRNA axis in the regulation of NSCLC cell growth, migration, and invasion *in vitro*

**Figure 4:**
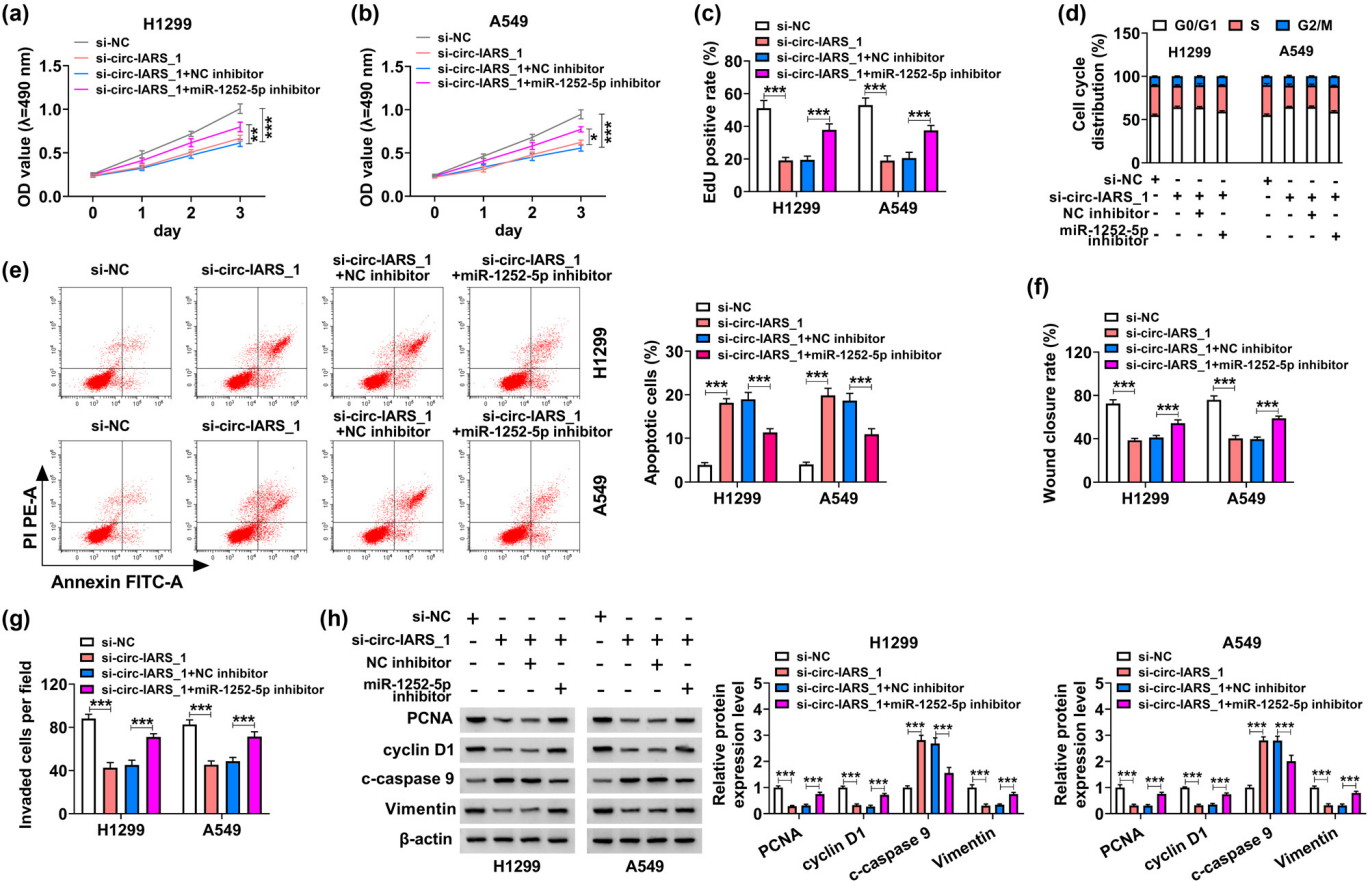
Downregulation of miR-1252-5p counteracted the effects of circ-IARS exhaustion in NSCLC cells. H1299 and A549 cells transfected with si-NC, si-circ-IARS_1, si-circ-IARS_1 + miR-1252-5p inhibitor, or si-circ-IARS_1 + NC inhibitor. (a and b) MTT assay monitored the OD value at 490 nm. (c) EdU assay determined the EdU-positive rate. (d) FCM method analyzed the cell cycle distribution (%) in G0/G1, S, and G2/M phases. (e) FCM method analyzed the apoptotic cells (%). (f) Scratch wound assay measured the wound closure rate. (g) Transwell assay examined the invaded cells per field (100×). (h) Western blotting detected the relative protein expression level of PCNA, cyclin D1, c-caspase 9, and vimentin in transfected cells, normalized to β-actin. *P < 0.05, **P < 0.01, and ***P < 0.001.

**Figure 5:**
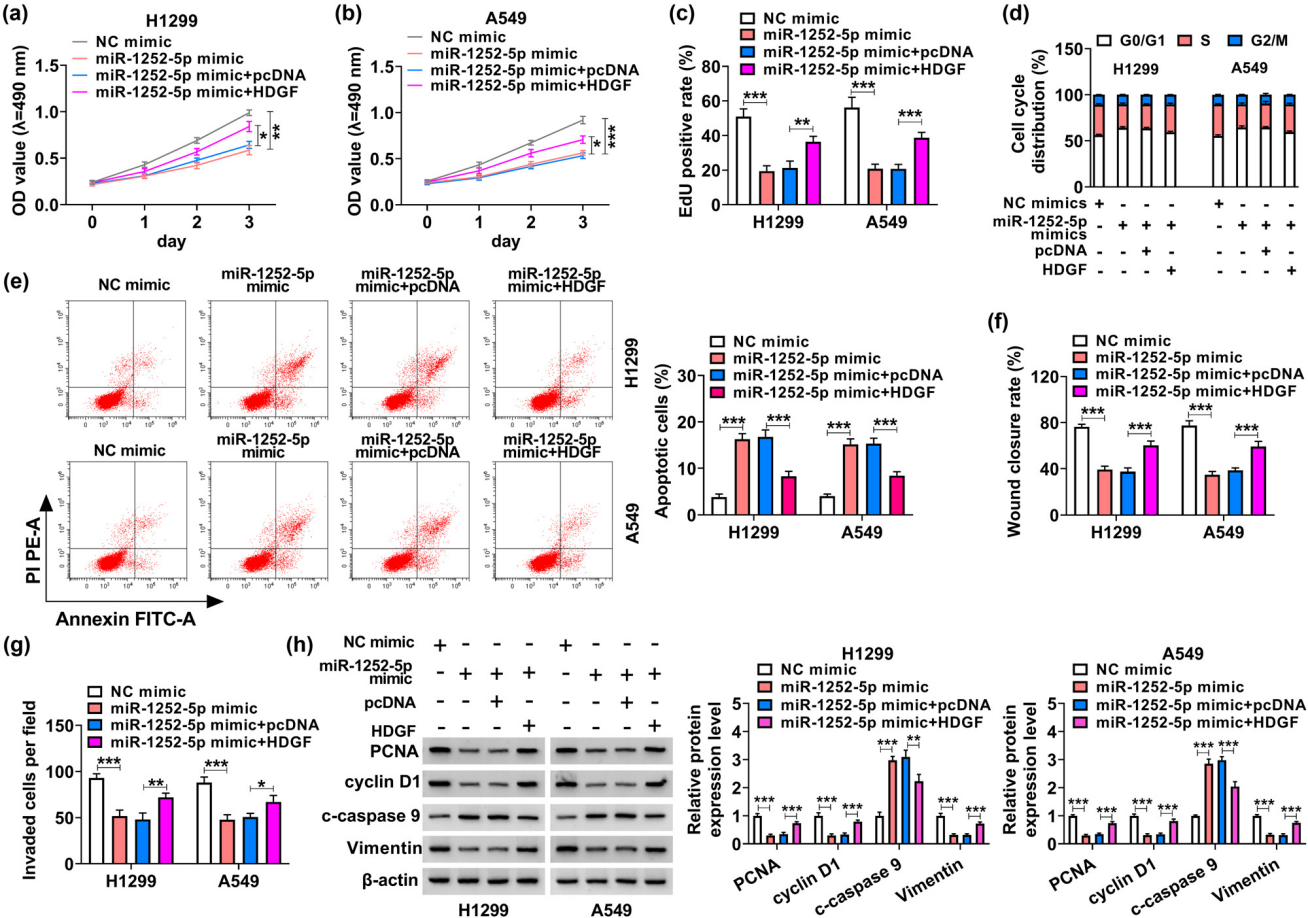
Overexpressing miR-1252-5p suppressed the development of NSCLC cells through HDGF. H1299 and A549 cells transfected with NC mimic, miR-1252-5p mimic, miR-1252-5p mimic + HDGF vector, or miR-1252-5p mimic + pcDNA vector. (a and b) MTT assay monitored the OD value at 490 nm. (c) EdU assay determined the EdU positive rate. (d and e) FCM method analyzed the cell cycle distribution (%) and apoptotic cells (%). (f) Scratch wound assay measured the percentage of wound closure rate. (g) Transwell assay examined the invaded cells per field (100×). (h) Western blotting detected the relative protein expression levels of PCNA, cyclin D1, c-caspase 9, and vimentin in transfected cells, normalized to β-actin. *P < 0.05, **P < 0.01, and ***P < 0.001.

Rescue experiments were carried out to measure the interactive effects between miR-1252-5p and circ-IARS or HDGF in NSCLC cells. Cell proliferation analyzed by MTT assay and EdU assay was inhibited by silencing circ-IARS (Figure 4a–c), and it could be similarly attenuated by overexpressing miR-1252-5p (Figure 5a–c); moreover, these anti-proliferative effects in H1299 and A549 cells could be severely mitigated by simultaneously transfecting miR-1252-5p inhibitor and HDGF vector (Figures 4a–c and 5a–c). Blocking circ-IARS arrested cell cycle of H1299 and A549 cells, and this arrested cell cycle progression was rescued by concurrently depleting miR-1252-5p, as indicated by more cells distributed in the S phase and fewer cells in G0/G1 phase (Figure 4d). Similar data were captured in H1299 and A549 cells transfected with miR-1252-5p mimic alone or together with HDGF vector (Figure 5d). Additional downregulation of miR-1252-5p abrogated the apoptosis promotion induced by circ-IARS deficiency (Figure 4e). Contrarily, upregulation of this miRNA via its mimic transfection elevated the percentage of apoptotic cells in H1299 and A549 cells, which was also then abated by additionally restoring HDGF (Figure 5e). Wound closure rate and invaded cells of H1299 and A549 cells could be suppressed by artificially exhausting circ-IARS or supplementing miR-1252-5p (Figures 4f, g and 5f, g), and this suppression was rescued in the co-presence of miR-1252-5p inhibitor and HDGF vector (Figures 4f, g and 5f, g). These results demonstrated that miR-1252-5p downregulation counteracted the tumor-suppressive role of circ-IARS exhaustion in NSCLC cells *in vitro*, and HDGF restoration abolished the anti-tumor role of miR-1252-5p upregulation in the malignant growth, migration, and invasion as well, suggesting the role of circ-IARS/miR-1252-5p/HDGF ceRNA axis in the regulation of NSCLC malignancy.

### 3.4 Silencing circ-IARS retarded tumor growth of NSCLC *in vivo* by upregulating miR-1252-5p and downregulating HDGF

**Figure 6:**
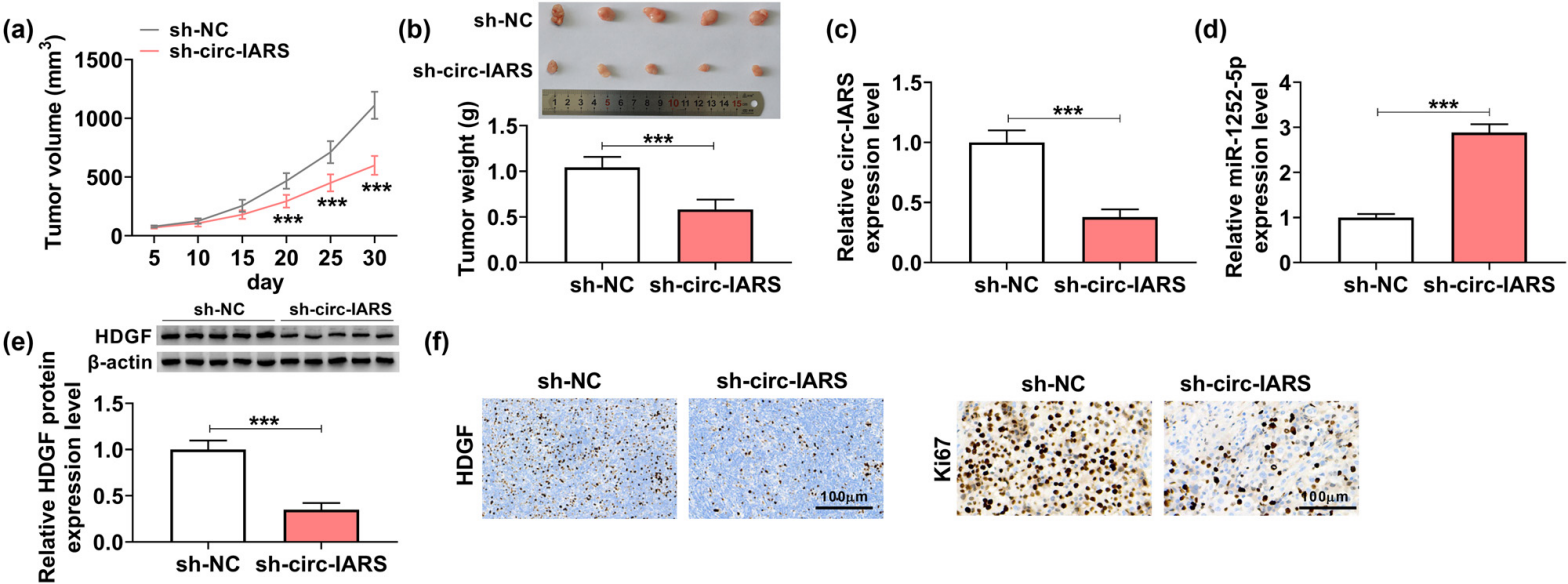
Silencing circ-IARS retarded the tumor growth of NSCLC *in vivo*. (a) Tumor volume was monitored every 5 days after inoculation of sh-circ-IARS or sh-NC-transfected H1299 cells into nude mice (*N* = 5). (b) Tumor weight was examined at the end day of the xenograft experiment. (c and d) RT-qPCR detected the relative circ-IARS and miR-1252-5p expression, and (e) western blotting measured the relative HDGF protein expression in tumor tissues from nude mice. (f) IHC examined the HDGF and Ki67 expression in paraffin-embedded xenograft tumor tissues. ***P < 0.001.

Furthermore, H1299 cells stably transfected with sh-circ-IARS or sh-NC were used to induce xenograft tumors in nude mice. After cell inoculation, tumor volume was monitored every 5 days, and tumors were smaller or lighter in the sh-circ-IARS group than in the sh-NC group (Figure 6a and b). Moreover, circ-IARS expression was reduced by 62.00% in xenograft tumor tissues than in the sh-circ-IARS group (Figure 6c), which was accompanied by 2.89-fold upregulation of miR-1252-5p and 65.00% downregulation of HDGF (Figure 6d and e). Besides, expression of HDGF and Ki67 was simultaneously inhibited in xenograft tumors (Figure 6f). These outcomes revealed a suppressive role of circ-IARS knockdown in tumor growth of NSCLC *in vivo* via regulating miR-1252-5p and HDGF.

### 3.5 Circ-IARS was upregulated in NSCLC patients’ tissues and serum exosomes

**Figure 7:**
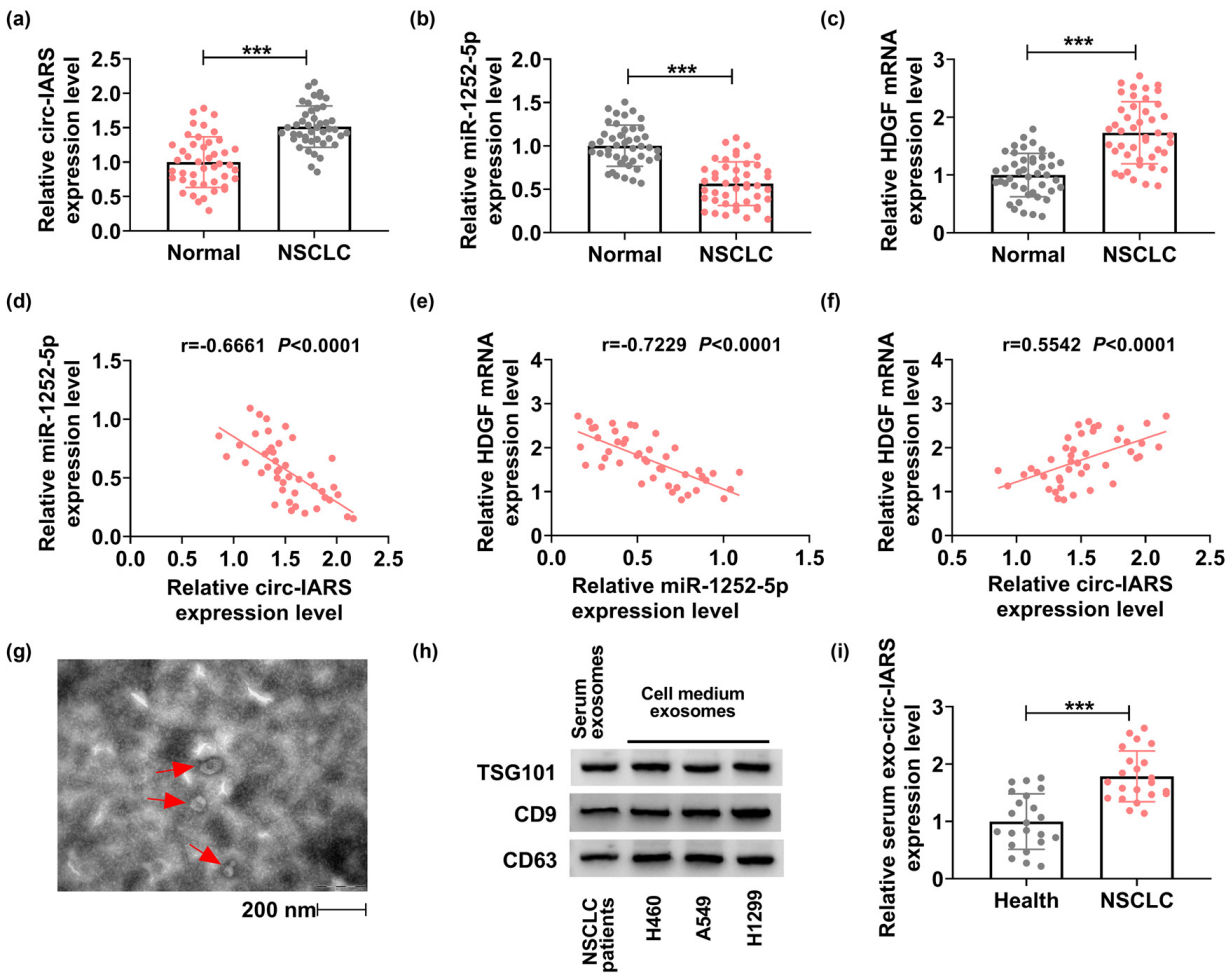
Circ-IARS was upregulated in NSCLC patients. (a–c) RT-qPCR detected the relative expression of circ-IARS, miR-1252-5p, and HDGF mRNA in NSCLC tumor tissues (NSCLC; *N* = 44), normalized to that in normal para-carcinoma tissues (Normal; *N* = 44). (d–f) Spearman’s rank correlation analysis determined the linear correlation among circ-IARS, miR-1252-5p, and HDGF mRNA expression in NSCLC tumors. (g) TEM showed the exosomes isolated from the serum of NSCLC patients. (h) Western blotting measured the expression of TSG101, CD9, and CD63 in isolated exosomes from NSCLC serum and NSCLC cell medium. (i) RT-qPCR detected the relative exo-circ-IARS expression in the serum of NSCLC patients (NSCLC; *N* = 22), normalized to serum from healthy people (Healthy; *N* = 22). ***P < 0.001.

Paired tissues were recruited from 44 NSCLC patients, and circ-IARS and HDGF were upregulated, while miR-1252-5p was downregulated in NSCLC tumor tissues (Figure 7a–c). In addition, miR-1252-5p expression in NSCLC tumors was negatively correlated with either circ-IARS or HDGF mRNA (Figure 7d and e), there was a positive correlation between circ-IARS and HDGF mRNA expression, as well (Figure 7f). Accidently, exosomes were isolated from NSCLC patients’ serum, and TEM showed a nearly spherical shape and size (Figure 7g). Markers of exosomes (TSG101, CD9, and CD63) were expressed in isolated exosomes from both NSCLC serum and cultured NSCLC cell medium, as indicated by western blotting (Figure 7h). Expression of circ-IARS was also detected in serum exosomes, and the results showed that exo-circ-IARS was highly expressed in the serum of NSCLC patients (*N* = 22) than in the serum of healthy people (*N* = 22) (Figure 7i). These results presented an upregulation of circ-IARS in NSCLC patients’ tissues and serum exosomes, suggesting that exo-circ-IARS has the potential as a diagnostic biomarker in NSCLC.

### 3.6 Exo-circ-IARS contributed to the proliferation, migration, and invasion of NSCLC cells *in vitro*

**Figure 8:**
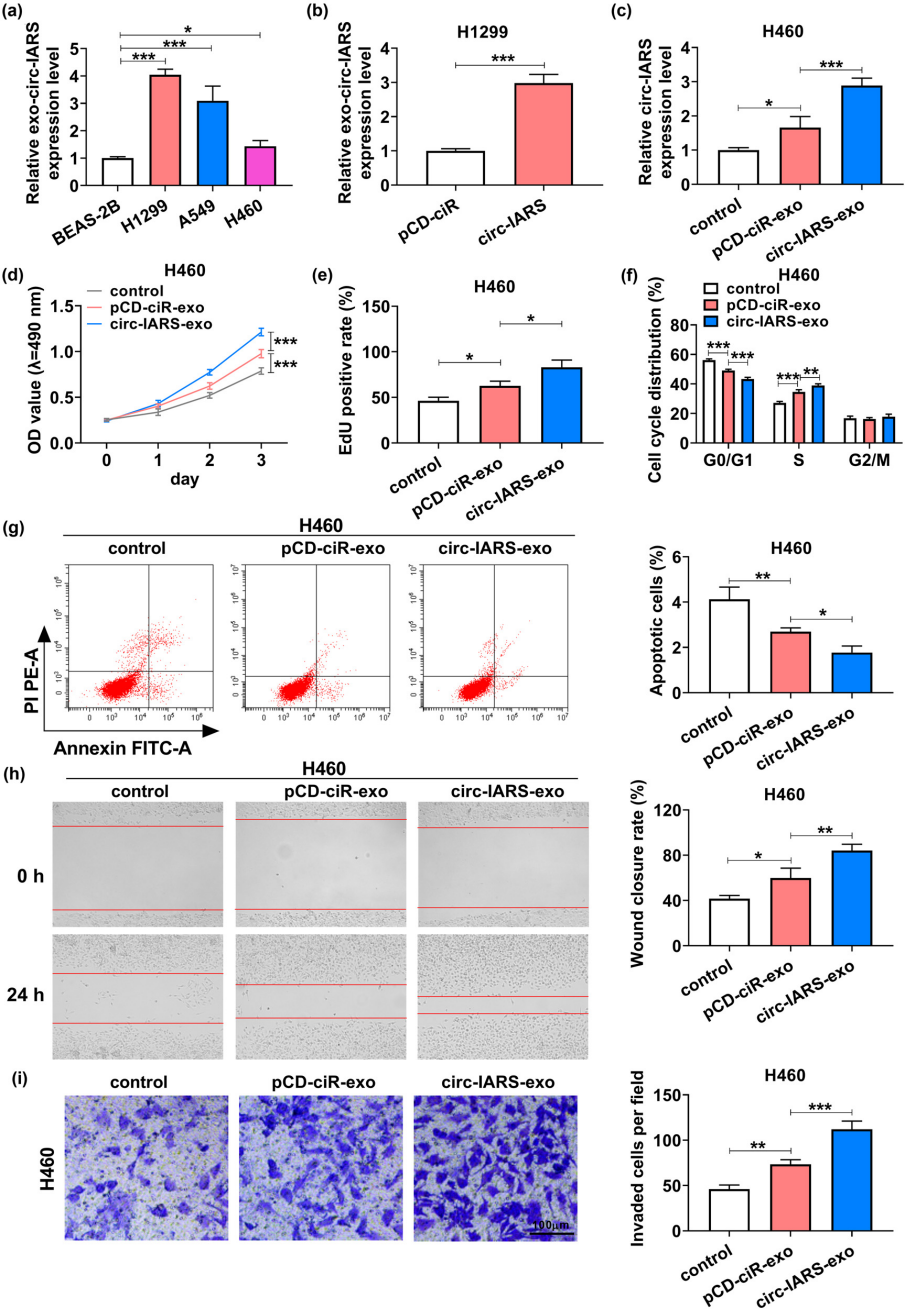
Ex-circ-IARS contributed to the growth, migration, and invasion of NSCLC cells *in vitro*. (a and b) RT-qPCR detected the relative exo-circ-IARS expression level in (a) BEAS-2B, H1299, A549, and H460 cells, and (b) H1299 cells transfected with circ-IARS or pCD-ciR vector. (c–i) H460 cells were co-cultured with H1299 cells-based exosomes after circ-IARS or pCD-ciR vector transfection (pCD-ciR-exo or circ-IARS-exo), normalized to control cells (without exosome treatment). (c) RT-qPCR detected the relative circ-IARS expression level. (d) MTT assay monitored the OD value at 490 nm. (e) EdU assay determined the EdU-positive rate. (f and g) FCM method analyzed the cell cycle distribution (%) and apoptotic cells (%). (h) Scratch wound assay measured the wound closure rate. (i) Transwell assay examined the number of invaded cells per field (100×). *P < 0.05, **P < 0.01, and ***P < 0.001.

Expression of exo-circ-IARS was overall more than 1.44 fold higher in the cell medium of NSCLC cell lines than that in BEAS-2B cells (Figure 8a). Among these NSCLC cells, exo-circ-IARS was highest in H1299 cells and was lowest in H460 cells (Figure 8a). Circ-IARS vector was transfected into H1299 cells to obtain a higher level of exo-circ-IARS, with a 2.98-fold change (Figure 8b). With co-culture with exosomes from H1299 cell medium, H460 cells showed an increase in circ-IARS expression in the pCD-ciR-exo group than in the control group, and a more increase (2.89 fold) in the circ-IARS-exo group (Figure 8c). Functionally, H460 cell proliferation was promoted by incubating with pCD-ciR-exo and was further promoted by incubating with circ-IARS-exo (Figure 8d–f). On the contrary, pCD-ciR-exo treatment could diminish the percentage of apoptotic cells with a change of 34.47%, and apoptotic cells were even reduced by 57.04% with circ-IARS-exo treatment (Figure 8g). In terms of cell motility, cell migration and invasion were augmented in pCD-ciR-exo-cultured H460 cells and were excessively augmented in circ-IARS-exo-cultured H460 cells (Figure 8h and i). These data demonstrated that NSCLC cell proliferation, migration, and invasion were promoted with the increase in circ-IARS level via exosome secretion, suggesting exo-circ-IARS contributed to malignant behaviors of NSCLC cells *in vitro*.

## 4 Discussion

Serum exosomes and several exosomal circRNAs have been identified as diagnostic biomarkers for NSCLC [17,21,22], lymph node metastasis, and chemoresistance in NSCLC [23,24]. In this study, we found that circ-IARS expression was increased in NSCLC cells, NSCLC tissues, and serum exosomes from NSCLC patients. Circ-IARS silencing inhibited NSCLC cell proliferation, migration, and invasion and promoted cell apoptosis. In terms of mechanism, circ-IARS combined with miR-1252-5p to induce the expression of the downstream gene HDGF. In addition, exo-circ-IARS promoted H460 cell proliferation, migration, and invasion and inhibited cell apoptosis. Silencing circ-IARS retarded tumor growth of NSCLC cells *in vivo*. Thus, circ-IARS, secreted by exosomes, contributed to NSCLC progression through circ-IARS/miR-1252-5p/HDGF pathway.

According to Gene Expression Omnibus database (GSE104854), circ-IARS was abnormally expressed in LUAD tumors than in paired non-tumor tissues. Consistent with that RNA sequencing data [14], we observed an upregulation of circ-IARS in this cohort of NSCLC patients’ tumors and cells; moreover, its expression was also higher in the exosomes from NSCLC patients’ serum and cell medium. Silencing circ-IARS via siRNA transfection suppressed the excessive proliferation, migration, invasion, and cell cycle progression of H1299 and A549 cells, as well as impeded tumor growth *in vivo*. Apoptosis of NSCLC cells *in vitro* was inversely controlled by circ-IARS expression. Promoting circ-IARS via exosome secretion facilitated the proliferation, migration, and invasion of H460 cells. These aforementioned outcomes implied similar patterns between circulating exosomal circRNA and tumor-derived circRNA. The phenomenon that circ-IARS was delivered from H1299 cells via exosomes to H460 cells suggested that exo-circ-IARS regulated tumor microenvironment and intercellular communication of NSCLC. This study considered that circ-IARS, secreted by exosomes, was an oncogenic driver in NSCLC via circ-IARS/miR-1252-5p/HDGF ceRNA axis. Furthermore, the difference in exo-circ-IARS expression in different NSCLC cell lines endowed itself a property of potential target for personalized treatment. Collectively, exo-circ-IARS might be a potent blood-based biomarker in NSCLC, and the relationship between exo-circ-IARS expression and clinical characteristics of NSCLC patients remained to be further confirmed.

Studies focusing on miR-1252-5p have been emerging and ongoing in human cancers in recent years, and miR-1252-5p could be sponged by different circRNAs. For example, hsa_circ_0011290 [25], hsa_circ_0000190 [26], and circ-ABCB10 [27,28] regulated proliferation, apoptosis, and migration of cancer cells through mode-of-action of sponging miR-1252-5p. Besides, glycolysis and chemoresistance were also affected by the circRNA/miR-1252-5p/mRNA crosstalk [26,29]. In NSCLC, miR-1252-5p was implicated in Cinnamaldehyde function against NSCLC via hsa_circ_0043256/miR-1252-5p/Itchy E3 ubiquitin protein ligase (ITCH) axis and Wnt/β-catenin signaling pathway [30]. Here expression of miR-1252-5p was downregulated in NSCLC patients’ tumors and cells, and restoring miR-1252-5p restrained proliferation and migration whereas enhanced apoptosis of NSCLC cells, which were in favor with the previous findings [28,30]. Additionally, cell cycle profile and invasion could also be altered by miR-1252-5p dysregulation in NSCLC, which might be first demonstrated in this study. A novel miR-1252-5p/HDGF interaction was further discovered in NSCLC cells and was the downstream target of circ-IARS.

HDGF was a well-recognized prognostic marker for patients with early-stage NSCLC, and it was correlated with poor overall, disease-specific, and disease-free survivals [31]. A previous study has revealed that HDGF silencing inhibited bladder cancer cell tumorigenesis and induced cell apoptosis through the PI3K-AKT signaling pathway [32]. Liang et al. also explained that HDGF combined with PI3K-AKT signaling pathway to promote colon cancer cell proliferation [33]. Antibody targeting HDGF was a novel effective strategy in treating lung cancers [19,34]. HDGF is also a pro-metastatic factor in NSCLC and promotes cell growth, motility, glycolysis, angiogenesis, and chemoresistance [35,36,37,38]. Here we noticed an upregulation of HDGF mRNA in NSCLC patients’ tumors and cells, and higher HDGF was associated with increased cell proliferation, cell cycle progression, migration, and invasion of NSCLC cells, as well as reduced apoptosis. MiRNAs including miR-16, miR-497, and miR-139-5p targeting HDGF in NSCLC cells participate in the malignant progression [35,37,39]. Moreover, Fu et al. [40] declared that suppression of HDGF/DEAD-Box Helicase 5 (DDX5)/β-catenin/c-Myc signaling could activate miR-296-3p in LUAD cell proliferation, metastasis, and chemoresistance. We identified a novel miR-1252-5p/HDGF interaction in NSCLC; however, the downstream targeted genes for HDGF need to be further analyzed, such as p53 [41] and DDX5 [40].

In conclusion, we showed that circ-IARS was upregulated in NSCLC patients and that circ-IARS and exo-circ-IARS synchronously contributed to cell growth, migration, and invasion of NSCLC via regulating miR-1252-5p/HDGF axis. This study suggested that exo-circ-IARS was a potential diagnostic biomarker for NSCLC and a potential target for the treatment of NSCLC. Interfering circ-IARS might be a promising therapeutic approach to NSCLC.

## Appendix

**Table A1:** Clinicopathological factors of NSCLC patients and healthy volunteers

Parameters	NSCLC (*n* = 44)	Healthy (*n* = 22)
**Age** (n, %)		
<60	25 (56.8%)	12 (54.5%)
≥60	19 (43.2%)	10 (45.5%)
**Gender** (n, %)		
Female	18 (40.9%)	9 (40.9%)
Male	26 (59.1%)	13 (59.1%)
**Body mass index** (kg/m^2^, mean ± SD)	23.28 ± 4.42	25.12 ± 5.10
**Smoking status** (n, %)		
Smoker	25 (56.8%)	12 (54.5%)
Non-smoker	19 (43.2%)	10 (45.5%)
**Histological subtype** (n, %)		
ADC	35 (79.5%)	
LCC	1 (2.3%)	
SCC	8 (18.2%)	
**Stage**		
I	2 (4.6%)	
II	14 (31.8%)	
III	28 (63.6%)	
**Lymph node metastasis** (n, %)		
Positive	21 (47.7%)	
Negative	23 (52.3%)	
**Tumor differentiation** (n, %)		
Well + moderate	24 (54.5%)	
Poor	20 (45.5%)	

ADC: lung adenocarcinoma; LCC: lung large-cell carcinoma; SCC: lung squamous carcinoma.
